# Clinical Evaluation of Maxillary Incisors Intrusion: Mini-Screws Versus Burstone Intrusion Arch

**DOI:** 10.1155/ijod/5004914

**Published:** 2025-11-24

**Authors:** Farzin Heravi, Maryam Omidkhoda, Alireza Chamani, Seyed Hossein Hosseini Zarch, Mohammadtaghi Shakeri, Benyamin Kazemi

**Affiliations:** ^1^Department of Orthodontics, School of Dentistry, Mashhad University of Medical Sciences, Mashhad, Iran; ^2^Department of Oral and Maxillofacial Radiology, School of Dentistry, Mashhad University of Medical Sciences, Mashhad, Iran; ^3^Department of Biostatistics, Faculty of Health, School of Health, Mashhad University of Medical Sciences, Mashhad, Iran

**Keywords:** Burstone intrusion arch, intrusion, mini-screw

## Abstract

**Objective:**

Maxillary incisors intrusion is needed in cases with overeruption of these teeth in deep bite and gummy smile. True intrusion requires precise mechanical planning and clinical supervision. This study compares the mini-screw (MS) and Burstone intrusion arch (BIA) methods clinically and radiographically to assess their effectiveness.

**Materials and Methods:**

Seventeen patients with deep bite were randomly assigned to two groups: the MS group (eight patients, aged 19.75 ± 2.48) treated with MS, and the BIA group (nine patients, aged 20.67 ± 5.41) treated with the BIA method. Ultra-low-dose CBCT (ULD CBCT) scans were taken at the start (T0), immediately after (T1), and 3 months after (T2) treatment. Intrusion amount, rate, inclination changes, overbite, gingival display, root resorption, and periodontal criteria were assessed at three-time intervals: T0–T1, T1–T2, and T0–T2. Data were analyzed using the independent *T*-test, with significance at *p*  < 0.05.

**Results:**

The MS group showed significantly greater intrusion than the BIA group at all three-time intervals (*p*  < 0.001). The active intrusion rate was also significantly higher in the MS method (*p*  < 0.001). No significant difference was found between the groups in the decrease of overbite. The BIA group showed some relapse during retention, whereas the MS group did not (*p*  < 0.001). The MS group also had a significantly greater decrease in gingival display than the BIA group (*p*  < 0.05).

**Conclusions:**

Both MS and BIA methods were effective for maxillary incisor intrusion. The MS method resulted in more true intrusion and less gingival display. After 3 months of retention, the MS group showed no vertical relapse, while the BIA group had a significant relapse of 0.1 mm (*p*  < 0.001).

**Summary**

• This clinical study compared maxillary anterior segment intrusion using mini-screws (MSs) and Burstone intrusion arch (BIA).
• The amount and rate of intrusion were significantly higher in the MS group.
• Both methods showed acceptable root resorption, which was not clinically significant.
• The MS method resulted in more true intrusion and less gingival display.
• The MS group showed no relapse, while the Burstone group had a slight relapse.
• Both methods increased pocket depth during active intrusion, but it was not clinically significant.
• The decrease in keratinized gingiva was significantly higher in the MS group during active intrusion.

## 1. Introduction

With the rapid development of skeletal anchorages in orthodontic treatment, clinicians have improved their ability to achieve favorable outcomes without worrying about anchorage loss [1]. Skeletal anchorages have also made it possible to accomplish many dental movements, such as intrusion [2, 3], which was not feasible with older treatments.

Four methods can correct deep bite, a common malocclusion in both children and adults: posterior teeth extrusion, flaring of the anterior teeth, intrusion of the incisors, and surgery in adult patients [4]. An untreated deep bite can lead to increased anterior crowding, maxillary dental flaring, periodontal problems, TMJ disorders, and issues with anterior and lateral mandibular movements [5–7]. Treatment selection depends on factors, such as the smile line, incisal show at rest, gingival display during a smile, and facial vertical height.

On the other hand, some patients with a deep bite also present with a gummy smile. Several treatment methods have been suggested to reduce a gummy smile, depending on factors, such as the lip-incisor relationship, lower facial height, clinical crown height of the teeth, gingival condition, lip height, and patient preferences. Some of the suggested treatment methods include; Lefort 1 osteotomy for maxillary impaction, upper lip lift surgery, injection of botulinum to reduce levator labii muscle tension, crown lengthening surgery, and maxillary anterior teeth intrusion [8–12].

Sometimes, a combination of these methods must be used to reduce gummy smile. One of the combined treatments to address both deep bite and gummy smile involves intruding the maxillary incisors in patients with normal vertical height [13]. Common methods for incisor intrusion include the utility arch, three-piece intrusion arch, and reversed curved arch. However, all three of these methods commonly result in anterior teeth tipping as a side effect [2, 14].

Temporary anchorage devices (TADs) can be used as a skeletal anchorage, allowing for better control of forces and potentially reducing the amount of root resorption associated with intrusive forces [15, 16]. The benefits of mini-screws (MSs) include immediate loading, the ability to place them in various locations in the mouth, such as interdental areas [16], the capability to apply a maximum force of 500 g to MS until the end of treatment without them becoming loose [17] and canine disimpaction [18, 19].

This clinical trial aimed to compare the traditional Burstone intrusion arch (BIA) method with an intrusion method using MSs. We considered the problems associated with common anterior intrusion methods and the potential benefits of using MS, especially in cases where true intrusion of the maxillary anterior segment is required. The clinical effects of MS in the maxillary anterior segment were evaluated.

## 2. Materials and Methods

### 2.1. Study Design

In this clinical trial (RCT code: IRCT20180929041176N1), 17 patients requiring intrusion of the maxillary anterior segment (equivalent to 68 teeth, including four maxillary incisors) were selected. The patients' ages ranged from 14 to 29 years. At the beginning of the study, written informed consent for participation and publication was obtained from all participants, and for participants under the age of 16, written consent was obtained from their parents or legal guardians. The inclusion criteria for this study were as follows: individuals who had passed the growth spurt (assessed through interview and cervical vertebral maturation), a skeletal Class I pattern, excessive gingival display, and an anterior deep bite requiring true intrusion of the maxillary anterior teeth, with an overbite ranging from 3.5 to 6.5 mm.

The exclusion criteria included any special medical condition, history of trauma, root resorption, previous orthodontic or orthopedic treatment, pathological lesions, and periodontal disease. Diagnostic evidence, including study casts, conventional photographs, panoramic radiographs, and lateral cephalometry, was collected before the start of treatment.

In all patients, the maxillary arch was divided into two segments: the anterior (incisors) and posterior (canine to first molar) segments. After segmental leveling and alignment from NiTi archwire to 16 × 22 stainless steel archwire were completed, the patients were randomly assigned to two groups in a 1:1 ratio generated by Microsoft Excel based on the type of intrusion. A full-size 18 × 25 stainless steel archwire was placed in both the anterior and posterior segments using the Roth 0.018-inch bracket system. The conventional BIA method was used to intrude the anterior segment in Group A (9 patients, 36 teeth), while two MSs were used in Group B (8 patients, 32 teeth).

Before and after the intrusion, and 3 months post-retention, photographs of the patients' posed smiles, as well as Planmeca Promax 3Dmax (Helsinki, Finland) and ultra-low-dose CBCT (ULD CBCT) radiographs, were obtained from the maxillary anterior segment. The following measurements were taken at the beginning of intrusion (T0), at the end of intrusion (T1), and after 3 months of retention (T2): amount of intrusion, linear root resorption, gingival display on smile, incisor inclination, and periodontal criteria. Changes in these criteria were calculated for three time periods: (T0–T1), (T1–T2), and (T0–T2). Data were collected and analyzed using statistical methods. It is important to note that no changes were made to the treatment of the mandibular arch throughout the intrusion treatment. Photographs of all patients were taken in the natural head position (NHP).

### 2.2. Group A: BIA Method

**Figure 1:**
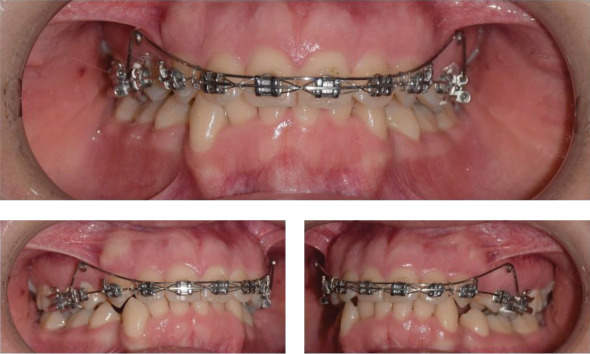
Intrusion of the anterior segment of the maxilla using Burstone's method.

The maxillary anterior and posterior arches were segmented. Triple bands with lingual sheaths were placed on the maxillary molars. A transpalatal arch (TPA) made with 0.9 mm (0.036˝) diameter wire was used to maximize posterior anchorage. The BIA, fabricated from a 16 × 22 stainless steel wire, was ligated distal to the lateral incisors at approximately the center of resistance (CR) of the anterior maxillary segment (Figure 1). The anterior segment was ligated using a ligature wire to apply force to the entire anterior segment. The intrusion force was set at 100 g and measured with a gauge. During the retention phase, a heavy continuous archwire was placed throughout the upper arch to prevent relapse. A step was inserted between the lateral and canine brackets to separate the anterior and posterior segments. The retention period lasted 3 months.

### 2.3. Group B: MS Method

**Figure 2:**
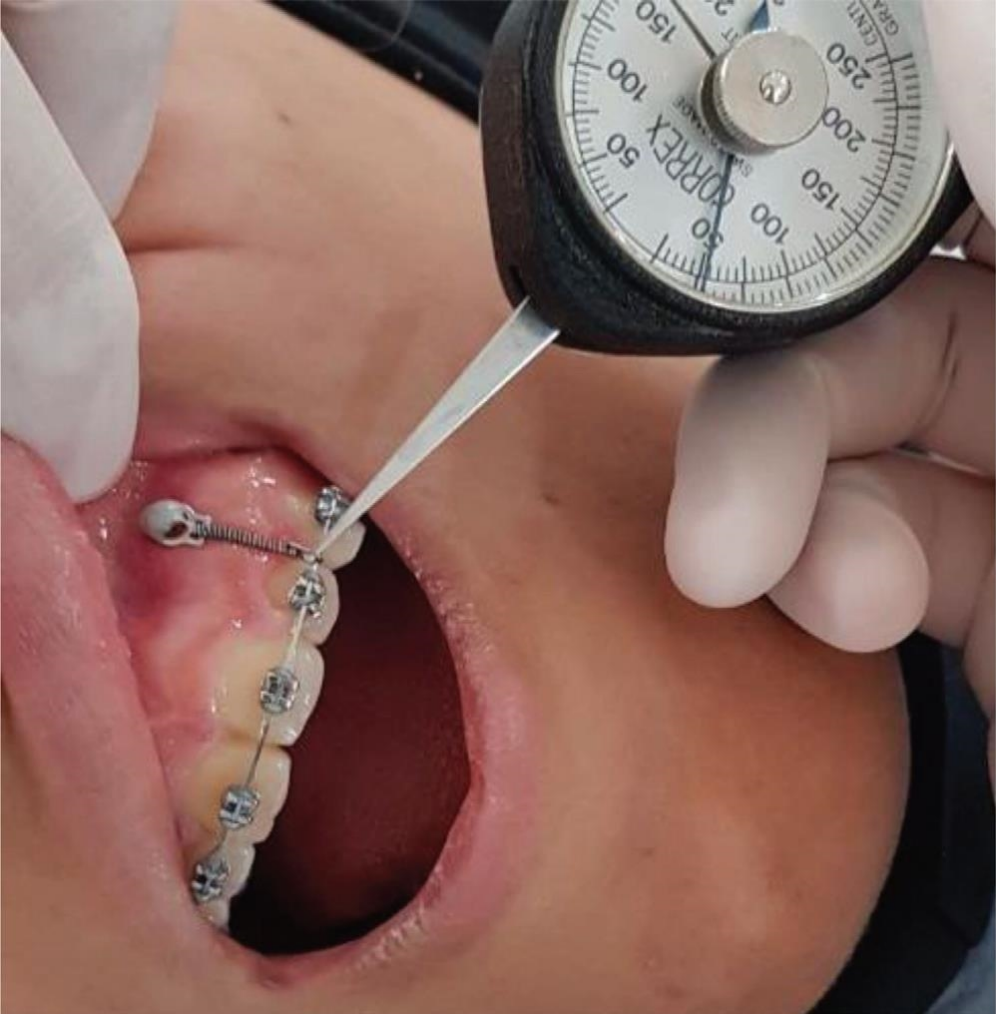
Measurement of intrusion force with a dynamometer in the mini-screw method.

After segmenting upper dental arch, MS (8 × 1.4 mm, G2, Dual top, Anchor System, Jeil Medical, Seoul, Korea) were placed in the area between the lateral incisor and canine roots (at the approximate location of the CR of the anterior maxillary segment). One week after placing the MS, a super-elastic closed coil spring was attached on each side to the bend of a sectional wire located distally to the lateral tooth. The force was measured using a force gauge (50 g on each side) (Figure 2).

While a full-dimension wire was located inside the brackets, the four maxillary incisors were ligated together with the ligature wire. Patients were recalled every 4 weeks for activation and to assess the amount of force. The maxillary incisor segment was securely attached to the MS with ligature wire after sufficient intrusion was achieved to reduce the possibility of relapse. This period lasted 3 months for all patients.

### 2.4. Periodontal Criteria Measurement

During the initial sessions before applying the force (T0), at the end of intrusion (T1), and after 3-month retention (T2), sulcus depth and the amount of keratinized gingiva were documented using a Williams periodontal probe. Additionally, the change in alveolar crest height at the CEJ was calculated using ULD CBCT radiography. The pocket depth was measured at four points (distobuccal, midbuccal, mesiobuccal, and midpalatal) using a periodontal probe. The average pocket depth at these four points was recorded at the beginning and end of intrusion and after 3-month retention. A decrease in pocket depth was documented with negative signs over time, whereas an increase was documented with positive signs.

### 2.5. Measuring Incisal and Gingival Display Changes in Smile

**Figure 3:**
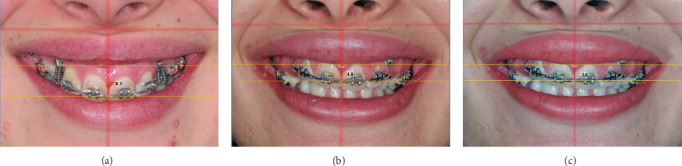
Measuring the reduction of incisal and gingival display in a smile. (a) T0, (b) T1, and (c) T2.

Photographs of posed smile were taken of all patients in a dedicated room using a professional camera (Nikon D50, Japan) mounted on a tripod 1 m away from the patient's face at the height of the patient's mouth. To ensure consistency, the patient's head was placed in a NHP before each stage. A vertical and horizontal laser line was used to ensure that the patient's head was in the same position for each photo, and the distance from the horizontal line to the canthus was measured. These steps were repeated three times (T0, T1, and T2) to evaluate changes in gingival and incisal display. To ensure accuracy, we measured the largest width of the upper right central maxillary incisor using radiography and Planmeca Romexis 5.3.4. To calculate changes in incisal and gingival display, we measured the vertical distance from the upper edge of the wire at the midline of the anterior segment to the lower edge of the upper lip when smiling. This measurement was taken three times (T0, T1, and T2) using Planmeca Romexis 5.3.4 software to obtain the true incisal and gingival display (Figure 3).

### 2.6. Measuring Radiographic Criteria

In the current study, ultra-low-dose imaging was used to capture radiographic landmarks three times. The radiographic characteristics were as follows: a 48 mm × 42 mm field of view (FOV; from the distal area of the maxillary lateral incisor on one side to the other side), 400-micron voxel, and 3 s exposure time with 55 DAP. All measurements were conducted using Planmeca Romexis (version 5.3.4) software with an accuracy of 0.01 mm at T0, T1, and T2.

**Figure 4:**
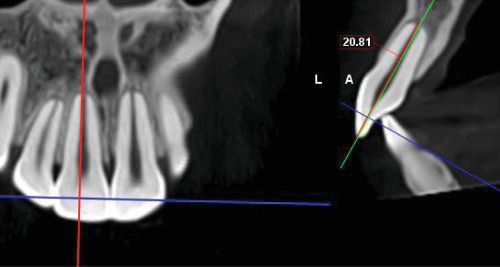
Measuring the maximum length of the tooth (sagittal view) to check the amount of linear root resorption.

**Figure 5:**
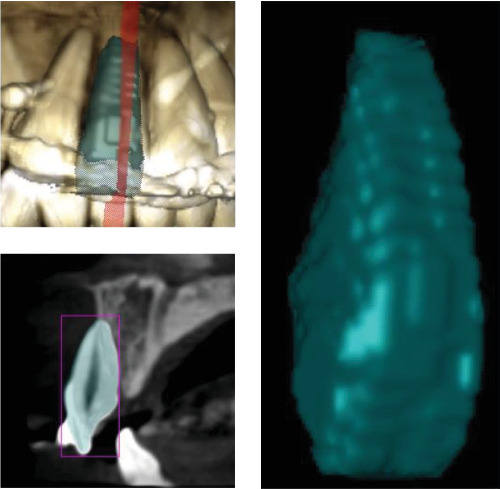
Measuring the maximum volume of the tooth to check the amount of volumetric root resorption.

**Figure 6:**
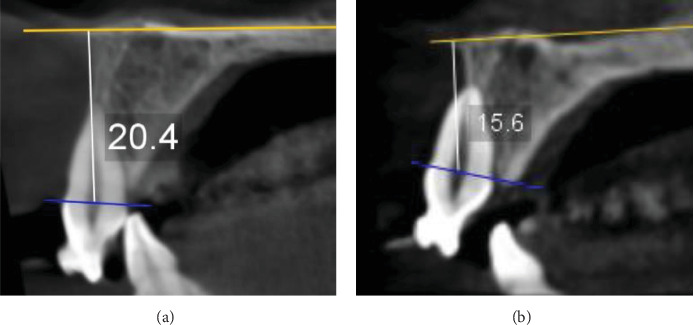
The vertical distance from the floor of the nasal cavity to the middle of the CEJ line connecting the buccal and lingual teeth of the central maxilla. (a) T0 and (b) T1.

**Figure 7:**
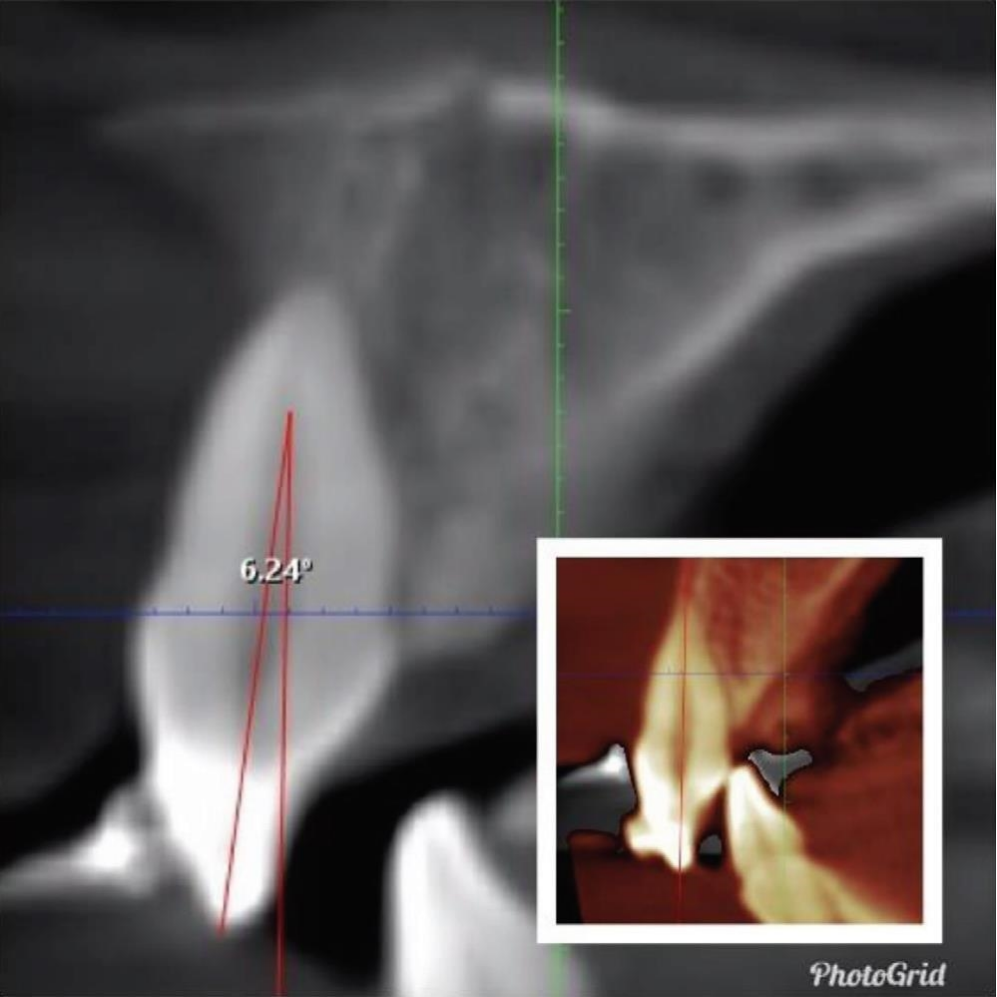
The rate of incisor inclination change with superimposition on the nasal floor in the ULD CBCT image.

**Figure 8:**
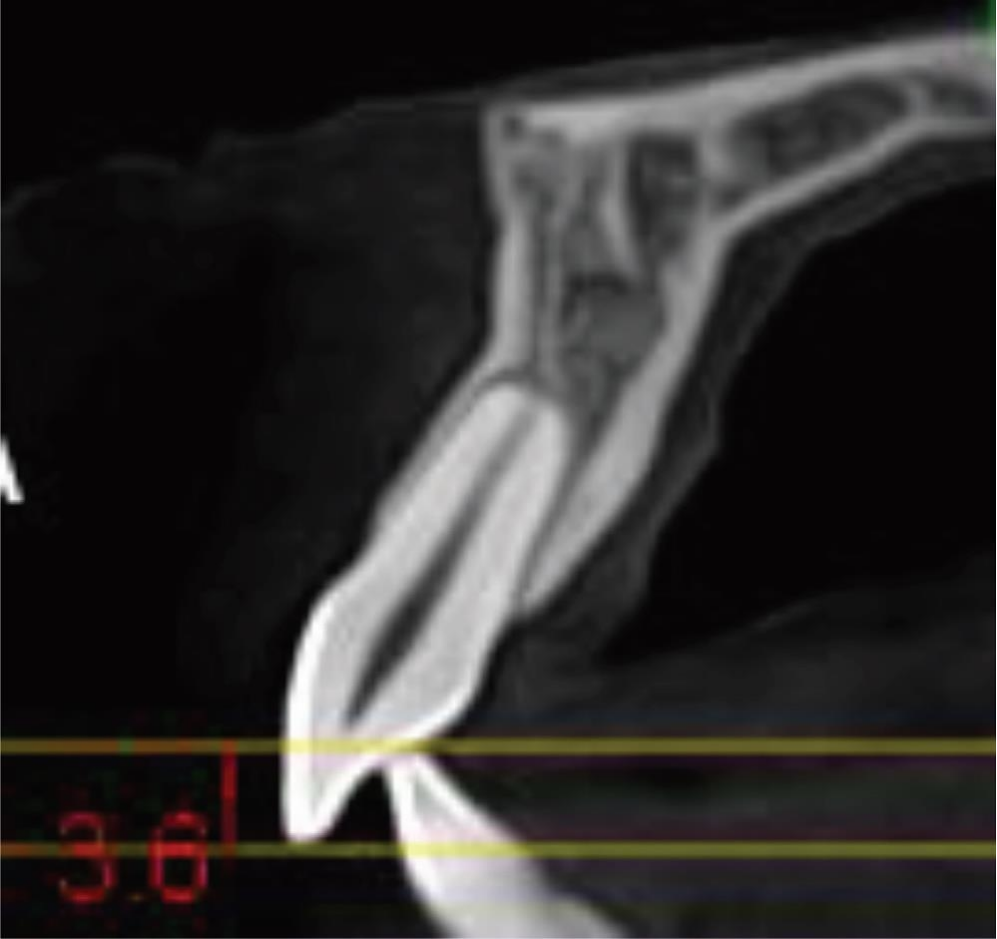
Measurement of overbite in the sagittal view of ULD CBCT radiography.

• Amount of linear root resorption: To calculate the amount of root resorption, the longest length of the tooth was measured in the sagittal view before and after intrusion and after 3 months of retention (Figure 4). The difference between these measurements indicated the amount of Linear root resorption at three specific periods: (T0–T1), (T1–T2), and (T0–T2).
• Amount of volumetric root resorption: To calculate the amount of volumetric root resorption, the tooth volume was measured before and after the intrusion, as well as after the retention period, using Planmeca Romexis software (Figure 5). The difference between these measurements indicated the amount of volumetric root resorption at three specific periods: (T0–T1), (T1–T2), and (T0–T2).
• Amount and rate of intrusion: The amount of intrusion was calculated by measuring the distance from the floor of the nasal cavity to the line connecting the buccal and lingual CEJ in each of the four incisors before and after the intrusion, as well as after the retention phase. Negative changes in these measurements indicated a decrease in distance and amount of intrusion, whereas positive changes indicated an increase in distance and potential treatment relapse (Figure 6). This process was repeated for all four incisors at each of the three phases, and the amount of intrusion at the three-time periods was calculated. The rate of intrusion per month was obtained by considering the number of months of active intrusion.
• Amount of incisal inclination change: In ULD CBCT radiography, the sagittal section was used to superimpose the radiography on the floor of the nasal cavity at two time points. A line was drawn along the longitudinal axis of the tooth to measure the angle. A positive angle change indicated the proclination of the teeth during the intrusion, whereas a negative angle indicated the uprighting of the tooth (Figure 7). This method was applied to each of the four incisors at the times—T0–T1, T1–T2, and T0–T2.
• Changes in the distance between the alveolar crest and the tooth's CEJ: To determine changes in the distance from the alveolar crest to the tooth's CEJ, the distance from the highest point of the crestal bone (the line connecting the mesial and distal alveolar crest of each tooth) to the line connecting the mesial and distal CEJ of each tooth at the three-time points of T0, T1, and T2 was measured.
• Amount of overbite change: In the ULD CBCT, sagittal sections were taken from the tip of the maxillary right incisor's crown. A line was drawn parallel to the horizon, and the amount of overlap at three different times (T0, T1, and T2) was calculated (Figure 8). A decrease in overbite was reported as negative, whereas an increase was reported as positive.

### 2.7. Statistical Methods and Sample Size

Data were analyzed using SPSS software (version 16) and appropriate statistical tests. The assumption of normality for all variables was confirmed using the one-sample Kolmogorov–Smirnov test at a 5% significance level. After confirming the normality of the data, an Independent *T*-test was used to assess the significance of changes in each variable.

The sample size was determined based on the study by Deguchi et al. [20], which included 15 participants in each group. However, due to the prevalence of COVID-19 at the time and the closure of educational facilities, such as universities, finding patients took longer than expected. Additionally, some patients were noncooperative in attending their follow-up visits on time. As a result, the required sample size was not reached.

### 2.8. Ethical Considerations

At the beginning of the study, written informed consent for participation and publication (including identifiable images in Figures 1–3) was obtained from all participants, and the advantages and disadvantages of each method were explained. For participants under the age of 16, written consent was obtained from their parents or legal guardians. Instead of conventional CBCT radiography, we used an advanced CBCT device called ULD CBCT, which has reduced radiography time, decreased FOV, and increased voxel size.

The research protocol of this study was prospectively registered with the RCT code IRCT20180929041176N1 on November 21, 2018, and approved by the Ethical Committee of Mashhad University of Medical Sciences (Number: IRMUMSDENTISTRY.REC.1397.101).

## 3. Results

The study involved 17 patients (16 females and 1 male), aged between 14 and 29 years. The average age of patients in the MS group was 19.75 ± 2.48 years, while in the BIA group, it was 20.67 ± 5.41 years (*p*=0.382). Using the MS method, 32 maxillary incisors were intruded, and using the BIA method, 36 maxillary incisors were intruded. Several measurements, such as linear root resorption, volumetric root resorption, amount of intrusion, rate of intrusion, changes in tooth angles, pocket depth changes, keratinized gingiva changes, incisal and gingival display changes, overbite changes, and changes in crest height to the tooth's CEJ were taken at three-time points: the start of intrusion (T0), the end of intrusion (T1), and 3 months retention (T2).

The normality of the data was assessed using the One-Sample Kolmogorov–Smirnov test for all variables, confirming normality at a significance level of 5%. Once the normality of the data was confirmed, an independent *T*-test was conducted to analyze the significance of the changes in each variable. In the tables, all variables were measured in millimeters, except for volumetric root resorption expressed in cubic centimeters, intrusion rate measured in millimeters per month, and angle changes measured in degrees.

Table 1 presents statistical information on data during the active intrusion phase of treatment (T0–T1). There was a significant difference in the average intrusion amount (*p*  < 0.001), active intrusion rate (*p*  < 0.001), average decrease in keratinized gingiva (*p*=0.025), and average decrease in gingival display (*p*=0.032) between the two methods. For the other variables, no statistically significant differences were found between the two methods (*p*  > 0.05).

The results from the intrusion retention phase (T1–T2) are presented in Table 2. In the MS method, the teeth experienced an average intrusion of 0.47 mm, while in the BIA method, there was a 0.1 mm relapse of intrusion, which was statistically significant (*p*  < 0.001). Additionally, overbite decreased by an average of 0.31 mm in the MS method, whereas in the BIA method, there was a 0.21 mm increase in overbite, which was also statistically significant (*p*  < 0.001). The two methods showed no statistically significant differences in the other variables (*p*  > 0.05).

The results obtained during the entire treatment time (T0–T2) are shown in Table 3. The difference in the average amount of intrusion (*p*  < 0.001), average pocket depth change (*p*=0.018), and average reduction in gingival display (*p*=0.016) between the two methods was statistically significant. For other variables, no significant statistical difference was observed between the two methods (*p*  > 0.05).

A comparison of active intrusion between the lateral incisors and central incisors revealed that the average intrusion in the central incisors was 1.82 mm, while the intrusion in the lateral incisors was 2.00 mm. No statistically significant difference in active intrusion between the lateral and central incisors was observed (*p*=0.561).

## 4. Discussion

In this study, 17 adult patients requiring intrusion of the maxillary anterior segment participated. After obtaining informed consent and assessing the patients' initial diagnostic documents and eligibility for the study, orthodontic treatment was initiated. In all patients, the maxillary arch was divided into two segments: an anterior segment (incisors) and a posterior segment (canine to first molar).

Following the completion of segmental leveling and alignment—progressing from NiTi archwires to 16 × 22 stainless steel archwires—participants were randomly allocated into two equal groups (1:1 ratio) using Microsoft Excel, based on the designated intrusion method. Subsequently, a full-size 18 × 25 stainless steel archwire was placed in both segments utilizing the Roth 0.018-inch bracket system. One group utilized two MS, while the other group used the conventional BIA for anterior segment intrusion.

Before and after the intrusion treatment, as well as 3 months after retention, photographs of the patients' posed smiles and ULD CBCT scans of the maxillary anterior segment were taken. This enabled the measurement of intrusion amount, linear and volumetric root resorption, gingival display, incisor intrusion, and periodontal criteria at three-time points: the start of intrusion (T0), the end of intrusion (T1), and 3-month after retention (T2). The changes in these criteria at the three-time intervals (T0–T1), (T1–T2), and (T0–T2) were then calculated, and the data were collected and analyzed statistically.

In our study, we found that the intrusion rate was higher with the MS method than with the BIA method. The greater decrease in keratinized gingiva and gingival display with the MS method can be attributed to a more true intrusion compared with the BIA method. This study confirms the greater efficiency of skeletal anchorage compared to dental anchorage during intrusion.

The average root resorption was less than 1 mm in both methods, and there was no statistically significant difference between volumetric and linear root resorption. This lack of difference can be attributed to the controlled application of the same force in both methods during each activation phase. Therefore, from a clinical perspective, both methods showed acceptable root resorption, which was not clinically significant.

During the active intrusion phase, both the MS and BIA methods increased the average pocket depth, possibly due to decreased oral hygiene in both groups during this phase. However, the increase was not clinically significant, as it was less than 0.5 mm. During the retention phase (T1–T2), different methods were used to prevent relapse of the intrusive treatment (prevention of extrusion). In the MS method, the anterior segment was tightly secured to the MS with a ligature wire, while a heavy sectional wire remained in the anterior segment. In the BIA method, a heavy continuous archwire was placed across the entire upper arch, with a step between the anterior and posterior segments (between the lateral and canine brackets).

Interestingly in retention phase (T1–T2), in the MS method, the teeth experienced an average intrusion of 0.47 mm. In contrast, the BIA method showed an average relapse of intrusion of 0.1 mm, which was statistically significant (*p*  < 0.001). In the BIA method, there was a possibility of relapse of the entire segment due to dental anchorage, although this was minimal (less than 0.1 mm) in our study.

The absence of relapse in the MS method can be attributed to the presence of skeletal anchorage, and greater intrusion can be achieved by applying a smaller force with the ligature wire connected to the MS during the retention phase.

Various studies have reported different MS placements for intruding the maxillary anterior segment. Based on an in vivo study [21], it was suggested that the CR for four maxillary incisors is located 8–10 mm more apically and 5–7 mm more distally than the lateral tooth. Therefore, the best placement for the MS is between the lateral and canine teeth in the most apical position possible. This placement is likely to result in the smallest change in the incisor angle during intrusion [22, 23]. For these reasons, in the present study, similar to most studies, the MS was placed between the lateral and canine teeth on both the right and left sides of the maxilla to apply force from the CR of the maxillary incisor segment.

Multiple studies have used different forces to intrude the maxillary anterior teeth. Burstone suggested using 80 g of force for the four upper incisors [23], while other studies have used forces ranging from 60 to 100 g [2, 14]. In this study, a force of 100 g was used for intruding maxillary incisors. Despite this higher force, the average amount of root resorption in all teeth was less than 1 mm, which is considered clinically acceptable. Unlike other studies in which root resorption was not assessed or only evaluated using periapical radiography at the start and end of the intrusion [14], this study provided a more thorough evaluation of root resorption. We used ULD CBCT with a limited FOV (48 mm × 42 mm) and a voxel size of 400 µm. The FOV selected for our study was even smaller than that used in Yeung's study [24]. Additionally, while the exposure time for a normal CBCT is 12–15 s, in our study, it was approximately 3 s.

In a study by Polat-Özsoy et al. [14], the effects of using MSs and Utility Arch for maxillary anterior segment intrusion were compared. The study found that the intrusion rate was 0.44 mm per month with the MS method and 0.27 mm per month with the utility arch method. The amount of intrusion with the MS method was 2.97 mm, whereas with the utility arch method, it was 1.81 mm. The study also noted that the amount of overbite reduction was 2.18 mm with the MS method and 2.32 mm with the utility arch method, though this difference was not statistically significant.

Additionally, the present study found the intrusion rate to be significantly higher with the MS method (0.64) than with the BIA method (0.29). During the active intrusion phase, the amount of overbite reduction was not statistically significant between the two methods (2.20 mm with MS and 2.38 mm with the BIA method). However, the amount of intrusion was significantly higher with the MS method (2.48 mm) than with the BIA method (1.14 mm). It seems that in both the present study and the study by Polat-Özsoy et al. [14], using the second method (BIA method in our study and utility arch in Polat-Özsoy's study) resulted in some overbite reduction as well as proclination of the incisors due to posterior segment extrusion.

One of the main benefits of NiTi superelastic springs is their ability to deliver an almost constant force across a wide range of deformation. When they are used, the force remains nearly stable throughout the tooth movement. Compared to conventional materials, these springs exhibit significantly less force degradation over time under constant extension [25]. In contrast, according to the force–deflection curve of the stainless steel wire, the force changes approximately linearly with deflection, and the elastic modulus of stainless steel is 3–5 times higher than that of the NiTi alloy [26, 27]. Therefore, it can be concluded that with a similar initial force, MS with NiTi closed coil springs can provide a more consistent light force compared to the stainless steel intrusion arch.

In our study, we aimed to prevent the BIA from affecting the posterior segment, as advised by Mr. Burstone. First, we leveled and aligned both segments to allow for the placement of a heavy rectangular wire [23]. Next, we placed a heavy sectional wire in the posterior segment and a TPA with a 0.9 mm diameter wire to minimize anterior intrusion impact. However, it seems that the true amount of intrusion differed from the overbite correction, suggesting a potential posterior extrusion in the BIA method. This discrepancy should be further investigated in future studies. Unfortunately, we could not measure posterior segment changes because we used a small FOV ULD CBCT limited to the incisor area.

Most studies on the retention phase did not evaluate intrusion in the anterior segment. In our study with the BIA group, a small relapse occurred over 3 months, while no relapse was observed in the MS method. This difference may be due to extrusive movements in the posterior segment during anterior intrusion in the BIA method, which could cause stretching of the masticatory muscles and the force exerted by muscle return during the retention phase [2], assuming that no changes occurred in the posterior segment with the MS method [2, 14].

In a study conducted by Kaushik et al. [28] in 2015, it was found that the use of MSs resulted in more intrusion than the two other methods, with no change in the axial inclination of the teeth. The overbite decreased in all three methods, especially in the MS group. In the present study, similar to the aforementioned study, minimal incisal proclination was observed in the MS group. However, contrary to the previous study, not much incisal proclination was observed in the BIA method.

In most studies that assessed the intrusion of anterior teeth, the periodontal condition of the intruded teeth was not considered, even though it seems to be very important in this type of tooth movement.

In our study, we found no clinically significant difference in pocket depth before, during, or after 3 months of retention, or throughout the entire treatment period. However, statistically, the increase in pocket depth over the whole treatment duration was greater in the MS group than in the BIA group. It is important to note that changes in pocket depth may become evident over the long term and require evaluation over longer periods. Additionally, the amount of keratinized gingiva significantly decreased only during the active intrusion phase in the MS method compared to that in the BIA method. However, in both methods, the decrease in the amount of keratinized gingiva was less than that in the amount of intrusion.

Overall, the use of MS for intrusion treatment has been shown to have no negative effects on the posterior segment. It can be used reliably and effectively to speed up the intrusion process, reduce gingival display, and achieve stability without adversely impacting periodontal health.

One of the limitations of this study was the relatively small sample size, as well as the inability to assess vertical, sagittal, and angular changes in the posterior segment for both treatment methods. This was due to the use of ULD CBCT with a small FOV, which was focused solely on the maxillary anterior region. It is recommended that future studies evaluate the stability of intrusion over a longer period (upto the completion of orthodontic treatment), include a larger sample size in each group, and assess changes in the posterior segment during the intrusion and retention phase to obtain more comprehensive and reliable results.

## 5. Conclusion

• The average amount and rate of intrusion in the MS method during the active phase of treatment and overall intrusive treatment was significantly higher than that in the BIA method and the MS method showed no signs of relapse.
• The average amount of linear root resorption in both methods was about 0.9 mm, and there was no statistically significant difference between the two methods during any of the periods.
• In both methods, there was less than a 5-degree proclination of the maxillary anterior segment, but no significant statistical difference was observed between the two methods.
• The amount of overbite reduction and intrusion continued during the retention phase in the MS group, whereas a slight increase in overbite was observed in the BIA method.
• In the MS method, during the active intrusion phase, there was a statistically significant reduction in gingival display and keratinized gingiva compared to the BIA method.

## Figures and Tables

**Table 1:** Statistical information of variables during the active intrusion phase of treatment (T0–T1).

Variables	Intrusion method	Mean	SD	Minimum	Maximum	*p*-Value
Linear root resorption	MS	−0.61	0.31	−0.12	−1.35	0.675
	BIA	−0.57	0.44	−0.09	−1.36	

Volumetric root resorption	MS	−0.045	0.021	−0.008	−0.088	0.111
	BIA	−0.036	0.014	−0.015	−0.073	

Amount of intrusion	MS	−2.48	1.08	−0.97	−4.91	<0.001*⁣*^*∗*^
	BIA	−1.14	0.77	−0.36	−3.09	

Rate of intrusion	MS	0.64	0.24	0.27	1.20	<0.001*⁣*^*∗*^
	BIA	0.29	0.11	0.12	0.15	

Incisal inclination change	MS	4.88	6.18	−12.23	13.86	0.64
	BIA	4.17	4.67	−8.26	8.73	

Pocket depth change	MS	0.4	0.66	−0.62	2.13	0.816
	BIA	0.36	0.37	−0.25	1	

Keratinized gingiva change	MS MS	−0.87	0.45	0.0	−1.5	0.025*⁣*^*∗*^
	BIA	−0.56	0.55	0.0	−1.5	

Gingival display change	MS	−2.35	1.17	−0.5	−4.30	0.032*⁣*^*∗*^
	BIA	−0.95	0.88	−0.2	−2.5	

Overbite change	MS	−2.20	0.64	−1.27	−3.20	0.698
	BIA	−2.38	1.04	−1.30	−4.20	

Alveolar crest change	MS	−0.08	0.32	0.6	−0.76	0.887
	BIA	−0.07	0.37	0.60	−1.04	

*⁣* ^*∗*^Represents statistical significance.

**Table 2:** Statistical information of variables during the intrusion retention phase (T1–T2).

Variables	Intrusion method	Mean	SD	Minimum	Maximum	*p*-Value
Linear root resorption	MS	−0.28	0.19	−0.04	−0.74	0.565
	BIA	−0.25	0.21	0.22	−0.83	

Volumetric root resorption	MS	−0.015	0.010	0.006	−0.043	0.285
	BIA	−0.018	0.012	0.004	−0.052	

Amount of intrusion	MS	−0.47	0.47	0.98	−1.92	<0.001*⁣*^*∗*^
	BIA	0.1	0.43	1.30	−0.45	

Incisal inclination change	MS	2.03	4.64	−4.80	14.37	0.909
	BIA	2.14	3.5	−4.27	10.64	

Pocket depth change	MS	0.01	0.45	−0.75	1.12	0.054
	BIA	−0.18	0.39	−1.00	0.87	

Keratinized gingiva change	MS	0.14	0.36	−0.5	1.00	0.209
	BIA	0.04	0.27	−0.5	0.5	

Gingival display change	MS	−0.31	0.24	0.00	−0.70	0.147
	BIA	−0.12	0.26	0.40	−0.60	

Overbite change	MS	−0.31	0.12	−0.15	−0.5	<0.001*⁣*^*∗*^
	BIA	0.21	0.19	−0.07	0.5	

Alveolar crest change	MS	0.13	0.38	−0.80	0.83	0.214
	BIA	0.03	0.22	−0.34	0.65	

*⁣* ^*∗*^Represents statistical significance.

**Table 3:** Statistical information of variables during the entire treatment time (T0–T2).

Variables	Intrusion method	Mean	SD	Minimum	Maximum	*p*-Value
Linear root resorption	MS	−0.9	0.36	−0.32	−1.87	0.582
	BIA	−0.84	0.33	−0.34	−1.59	

Volumetric root resorption	MS	−0.060	0.025	−0.004	−0.108	0.185
	BIA	−0.051	0.019	−0.020	−0.088	

Amount of intrusion	MS	−2.95	1.16	−1.34	−5.56	<0.001*⁣*^*∗*^
	BIA	−0.92	0.77	0.43	−2.77	

Incisal inclination change	MS	6.91	3.95	−2.00	13.11	0.482
	BIA	6.11	4.54	−3.64	12.28	

Pocket depth change	MS	0.4	0.6	−0.38	1.88	0.018*⁣*^*∗*^
	BIA	0.07	0.32	0.62	−0.63	

Keratinized gingiva change	MS	−0.73	0.47	0.00	−1.50	0.185
	BIA	−0.56	0.47	0.00	−1.50	

Gingival display change	MS	−2.66	1.21	−0.70	−4.70	0.016*⁣*^*∗*^
	BIA	−0.98	0.95	0	−2.60	

Overbite change	MS	−2.51	0.61	−1.62	−3.49	0.351
	BIA	−2.07	0.62	−0.85	−4.00	

Alveolar crest change	MS	0.07	0.39	0.59	−1.00	0.302
	BIA	−0.03	0.36	0.64	−0.88	

*⁣* ^*∗*^Represents statistical significance.

## Data Availability

The data used to support the findings of this study are included within the article.
